# *Hfe* Deficiency Impairs Pulmonary Neutrophil Recruitment in Response to Inflammation

**DOI:** 10.1371/journal.pone.0039363

**Published:** 2012-06-21

**Authors:** Karolina Benesova, Maja Vujić Spasić, Sebastian M. Schaefer, Jens Stolte, Tomi Baehr-Ivacevic, Katharina Waldow, Zhe Zhou, Ursula Klingmueller, Vladimir Benes, Marcus A. Mall, Martina U. Muckenthaler

**Affiliations:** 1 Department of Pediatric Oncology, Hematology and Immunology, University Hospital of Heidelberg, Heidelberg, Germany; 2 Molecular Medicine Partnership Unit, Heidelberg, Germany; 3 European Molecular Biology Laboratory (EMBL), Heidelberg, Germany; 4 Systems Biology of Signal Transduction, German Cancer Research Center (DKFZ), DKFZ-ZMBH-Alliance, Heidelberg, Germany; 5 Department of Translational Pulmonology, University of Heidelberg, Heidelberg, Germany; 6 Translational Lung Research Center Heidelberg (TLRC), Member of the German Center for Lung Research, Heidelberg, Germany; French National Centre for Scientific Research, France

## Abstract

Regulation of iron homeostasis and the inflammatory response are tightly linked to protect the host from infection. Here we investigate how imbalanced systemic iron homeostasis in a murine disease model of hereditary hemochromatosis (*Hfe^−/−^* mice) affects the inflammatory responses of the lung. We induced acute pulmonary inflammation in *Hfe^−/−^* and wild-type mice by intratracheal instillation of 20 µg of lipopolysaccharide (LPS) and analyzed local and systemic inflammatory responses and iron-related parameters. We show that in *Hfe^−/−^* mice neutrophil recruitment to the bronchoalveolar space is attenuated compared to wild-type mice although circulating neutrophil numbers in the bloodstream were elevated to similar levels in *Hfe^−/−^* and wild-type mice. The underlying molecular mechanisms are likely multifactorial and include elevated systemic iron levels, alveolar macrophage iron deficiency and/or hitherto unexplored functions of *Hfe* in resident pulmonary cell types. As a consequence, pulmonary cytokine expression is out of balance and neutrophils fail to be recruited efficiently to the bronchoalveolar compartment, a process required to protect the host from infections. In conclusion, our findings suggest a novel role for *Hfe* and/or imbalanced iron homeostasis in the regulation of the inflammatory response in the lung and hereditary hemochromatosis.

## Introduction

An active cross-talk between the regulation of cellular and systemic iron homeostasis and the immune response has evolved to protect the host from infections. A key innate immune defense mechanism is to limit iron availability for invading bacteria by retaining iron in macrophages. Inflammatory or infectious cues stimulate expression of the hepatic peptide hormone hepcidin, an acute phase protein and critical regulator of systemic iron homeostasis [1]. Hepcidin binds to the iron exporter ferroportin and triggers its internalization and degradation to limit iron release from duodenal enterocytes and macrophages [2], [3]. If the inflammatory stimulus persists, systemic iron deficiency will lead to the anemia of inflammation, a frequent disorder of hospitalized patients [1]. Conversely, the impact of disturbed iron homeostasis on the immune response of the host still raises many questions.

Hereditary hemochromatosis (HH) is a frequent genetic disorder characterized by intestinal iron hyperabsorption, hyperferremia and tissue iron accumulation [4]. The most common form of HH is associated with mutations in the *Hfe* gene, which encodes for an atypical MHC class I-like molecule [4], [5]. Mice homozygous for the *Hfe* null allele recapitulate the phenotype observed in humans with attenuated hepatic hepcidin expression and systemic iron overload [6], [7], [8]. Low hepcidin levels fail to inhibit ferroportin-controlled iron export from duodenal enterocytes and macrophages. As a consequence reticuloendothelial cells are iron deficient [6], [7], [8] as has been demonstrated for peritoneal macrophages [9], [10], [11] and Kupffer cells [12] in *Hfe^−/−^* mice.

In *HFE*-HH patients the immune response is altered and increased susceptibility towards extracellular pathogens on the one hand [13] and relative resistance to macrophage-associated intracellular bacteria on the other hand are reported [14]. Even more interesting, murine *Hfe*-deficiency is associated with an attenuated inflammatory response to *Salmonella* infection *in vitro* and *in vivo* [9], [10], [11]. The attenuated recruitment of immune cells to the site of inflammation has been attributed to reduced cytokine release from *Hfe*-deficient macrophages [9], [10], [15]. Subsequently, impaired TLR-4-signaling involving the TRIF (Tir domain-containing adaptor inducing interferon-beta (Ticam1))/TRAM (Trif-related adaptor molecule (Ticam2)) step has been proposed as the underlying mechanism and suggested to be caused by intracellular iron deficiency of *Hfe*-deficient macrophages [10]. By contrast, no alterations in cytokine release in *Hfe^−/−^* mice were reported following intraperitoneal LPS-administration [16] and in a second study of *Salmonella* infection [11]. The increased resistance of *Hfe^−/−^* mice to bacteraemia was therein associated with enhanced production of lipocalin-2 (Lcn2), an enterochelin-binding peptide involved in the innate immune response [11], [17].

The aim of this study was to investigate whether *Hfe* deficiency affects the inflammatory response of the lung induced by intratracheal instillation of LPS as a model of gram negative bacterial infection [18]. The lung is of particular interest because its constant exposure to airborne iron particles and pathogens must have led to potent mechanisms for iron detoxification and antimicrobial defense [19], [20]. In a healthy lung, iron homeostasis is kept in tight balance [19], [21]. However, this balance is prone to be disturbed by various exogenous and endogenous factors, including common ones such as cigarette smoke and particle exposure [21]. Elevated iron levels have been demonstrated in several acute and chronic diseases of the lung such as pneumonia and cystic fibrosis [19], [22]. Furthermore, increased availability of iron as a nutrient for pathogens promotes an ongoing pulmonary infection and subsequent inflammation [19], [22]. Conversely, there is little insight into how a primarily disturbed iron homeostasis and/or *Hfe* deficiency affect the pulmonary inflammatory response.

In this study, we induced an acute pulmonary inflammation in *Hfe^−/−^* and wild-type (WT) mice by intratracheal instillation of LPS and analyzed parameters of inflammation and iron homeostasis. Our data show that the LPS-triggered inflammatory response that is hallmarked by neutrophil [polymorphonuclear leukocytes] recruitment to the bronchoalveolar space [23], [24], [25] is significantly attenuated in *Hfe^−/−^* mice. Elevated systemic iron levels, alveolar macrophage iron deficiency and/or hitherto unexplored functions of *Hfe* in resident pulmonary cell types are expected to cause dysregulated pulmonary cytokine expression, which may be causative for the attenuated neutrophil recruitment in *Hfe^−/−^* mice. Collectively, our results provide novel insights into the role of Hfe in the regulation of the inflammatory response of the lung and the consequences of *Hfe*-deficiency and/or imbalanced iron homeostasis for HH-patients.

## Results

### LPS-induced Neutrophil Recruitment to the Lung is Attenuated in *Hfe^−/−^* Mice

**Figure 1:**
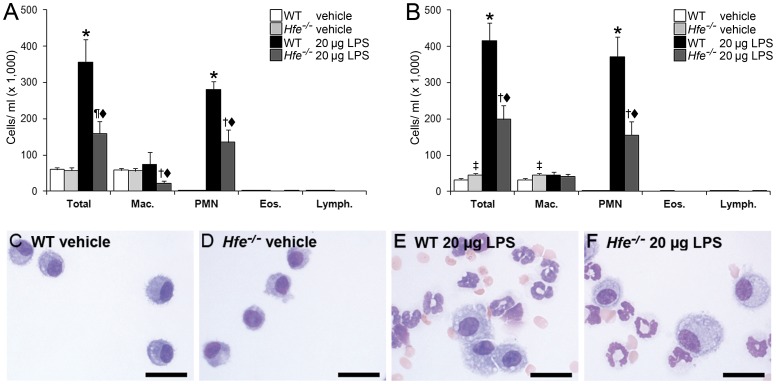
Attenuated inflammatory cell counts in the BAL of wild-type and *Hfe^−/−^* mice. BAL obtained 4 h after intratracheal instillation of vehicle or 20 µg LPS. (A) Female wild-type and *Hfe^−/−^* mice. n = 5–7 per group. ^★^ *P*<0.001 versus WT control mice; ^¶^ *P*<0.05 and ^†^ *P*<0.001 versus *Hfe^−/−^* control mice; ^⧫^ *P*<0.005 versus LPS-treated WT mice. (B) Male wild-type and *Hfe^−/−^* mice. n = 9–11 per group. ^★^ *P*≤0.001 versus WT control mice; ^‡^ *P*<0.05 and ^†^ *P*<0.005 versus *Hfe^−/−^* control mice; ^⧫^ *P*<0.005 versus LPS-treated WT mice. Mac.  =  macrophages; PMN  =  polymorphonuclear leukocytes/neutrophils; Eos.  =  eosinophils; Lymph.  =  lymphocytes. (C–F) Representative images of BAL cytospin slides obtained from WT and *Hfe^−/−^* mice (females). MayGrünwald-Giemsa stain, images obtained at 400× magnification. Scale bars, 20 µm.

To induce an acute pulmonary inflammation in the mouse we applied 20 µg LPS by intratracheal instillation and analyzed inflammatory cell counts in the bronchoalveolar lavage fluid (BAL) 4 h later. LPS instillation induced a considerable increase of total cell numbers in the BAL compared to vehicle-instilled wild-type mice, which was mainly attributed to neutrophil recruitment. The numbers of alveolar macrophages (AM) remained unchanged by LPS instillation and were comparable to the vehicle-instilled wild-type mice that almost exclusively contained macrophages. As expected, eosinophils and lymphocytes were only sporadically detected in the BAL independent of LPS instillation (Fig. 1A–D). Comparable results were obtained by pulmonary instillation of a lower LPS dose (1 µg) in wild-type mice (data not shown).

We next tested the effects of intratracheal LPS instillation in *Hfe* ^−/−^ mice. Similar to wild-type mice, the BAL of vehicle-instilled *Hfe^−/−^* mice almost exclusively contained macrophages and LPS instillation increased pulmonary neutrophil recruitment (Fig. 1A,B,E,F). Interestingly, both total cell counts and neutrophil numbers were significantly reduced by approximately 50% in LPS-treated *Hfe* ^−/−^ mice of both sex compared to LPS-treated wild-type mice (Fig. 1).

### LPS-induced Neutrophil Recruitment to the Bloodstream does not differ between *Hfe^−/−^* and Wild-type Mice

**Figure 2:**
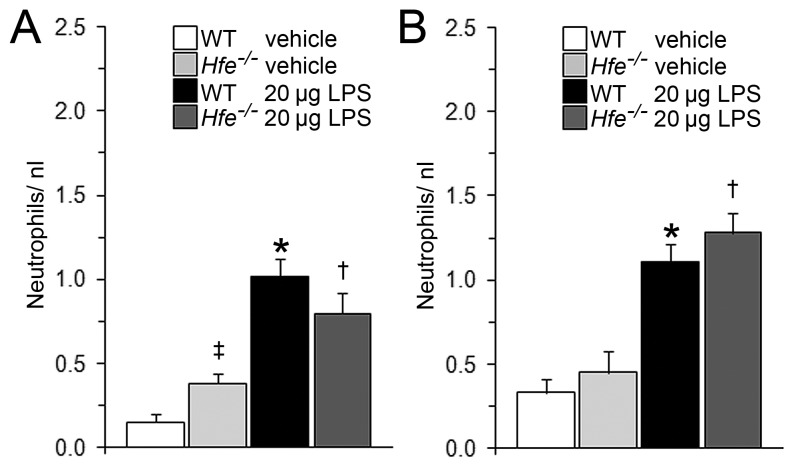
Circulating neutrophil levels (in cells/nL) in wild-type and Hfe^−/−^ mice. Blood was obtained 4 h after intratracheal instillation of vehicle or 20 µg LPS. (A) Female wild-type and *Hfe^−/−^* mice. n = 5–7 per group. ^‡^ *P*<0.05 and ^★^ *P*<0.001 versus WT control mice; ^†^ *P*<0.05 versus *Hfe^−/−^* control mice. (B) Male wild-type and *Hfe^−/−^* mice. n = 9–11 per group. ^★^ *P*<0.001 versus WT control mice; ^†^ *P*<0.001 versus *Hfe^−/−^* control mice.

Pulmonary LPS instillation triggers a systemic inflammatory response [26], [27], [28]. We therefore analyzed whether a reduced recruitment of neutrophils to the bloodstream in *Hfe^−/−^* mice could explain the attenuated recruitment of neutrophils to the lung. LPS instillation increased the number of circulating neutrophils to similar levels in *Hfe^−/−^* and wild-type mice (Fig. 2). This suggests that attenuated neutrophil recruitment to the lung of LPS instilled *Hfe^−/−^* mice are not a consequence of lower neutrophil numbers in the blood. We speculate that reduced recruitment of neutrophils to the lungs of *Hfe^−/−^* mice may either be a consequence of so far unexplored immune functions of the MHC class 1-like protein Hfe in macrophages or pulmonary cell types or by the alterations of systemic iron homeostasis that hallmark *Hfe^−/−^* mice.

### LPS-induced Neutrophil Recruitment does not Exclusively depend on *Hfe* Expression in Macrophages and Neutrophils

**Figure 3:**
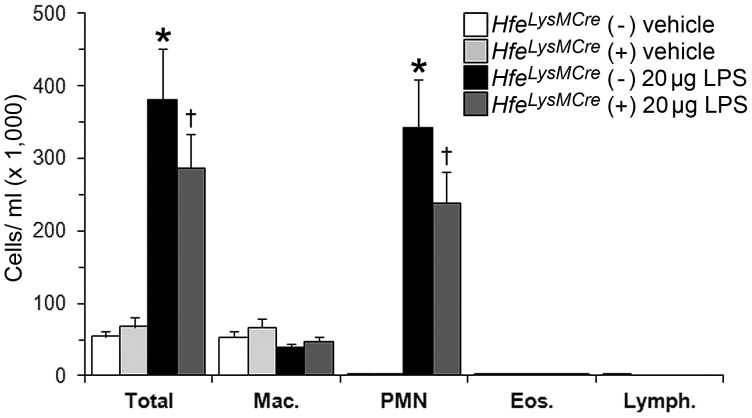
Inflammatory cell counts in the BAL of *Hfe^LysMCre^* mice. BAL obtained 4 h after intratracheal instillation of vehicle (n = 4–5 per group) or 20 µg LPS (n = 9–15 per group). ^★^ *P*≤0.005 versus *Hfe^LysMCre^* (−) control mice; ^†^ *P*≤0.005 versus *Hfe^LysMCre^* (+) control mice. Mac.  =  macrophages; PMN  =  polymorphonuclear leukocytes/neutrophils; Eos.  =  eosinophils; Lymph.  =  lymphocytes.

To investigate whether a lack of *Hfe* in macrophages/neutrophils explains reduced neutrophil recruitment to the lung of *Hfe^−/−^* mice, we analyzed the LPS-response in macrophage/neutrophil-*Hfe* deficient mice (*Hfe^LysMCre^* mice) [6]. Similar to *Hfe^−/−^* mice (Fig. 1A), LPS instillation induced a significant increase in total BAL cells and neutrophil numbers in both *Hfe^LysMCre^* (+) and their control *Hfe^LysMCre^* (−) mice. In contrast to *Hfe^−/−^* mice, LPS-treated *Hfe^LysMCre^* (+) showed partially reduced total BAL cell counts and neutrophil numbers (∼25%) compared to control mice, however without statistically significant difference (Fig. 3). Circulating blood neutrophils also increase to similar levels in *Hfe^LysMCre^* (+) and *Hfe^LysMCre^* (−) mice (Fig. S1). These data indicate that LPS-induced neutrophil recruitment does not exclusively depend on *Hfe* expression in macrophages/neutrophils and suggest that *Hfe* expression in other cell types in the lung (such as alveolar epithelia, endothelia or fibroblasts) and/or increased systemic iron levels may play a role in neutrophil recruitment.

### Effect of LPS-instillation on Iron Metabolism

We next analyzed iron-related parameters (plasma and tissue iron levels, hepatic hepcidin mRNA expression) in LPS-instilled female wild-type, *Hfe^−/−^, Hfe^LysMCre^* (−) and *Hfe^LysMCre^* (+) mice. *Hfe^LysMCre^* mice were included for further analyses as these may give indications about the trend towards lower neutrophil recruitment observed in this mouse line. Interestingly, pulmonary LPS-instillation did not result in generally reduced plasma iron levels (Table 1), despite increased hepatic hepcidin mRNA expression (Fig. S2). This result contrasts previous studies where upon intraperitoneal LPS administration a significant drop in plasma iron levels correlated with increased hepatic hepcidin expression [16], [29]. However, by comparison, the intratracheal LPS instillation likely produced substantially lower systemic LPS levels and was less efficient in inducing a hepatic hepcidin response. In addition, the iron content of the lung, the spleen and the liver remained largely unaffected following LPS instillation in wild-type and *Hfe^−/−^* mice and mildly increased in the liver of *Hfe^LysMCre^* (+) mice (Table 1). Thus, we propose that elevated plasma and liver iron levels present in *Hfe^−/−^* may be a factor contributing to attenuated pulmonary neutrophil recruitment.

**Table 1:** Plasma iron and non-heme tissue iron content in female wild-type, *Hfe^−/−^* and *Hfe^LysMCre^* mice.

	Experimental group	Plasma iron	Lung iron	Liver iron	Spleen iron
**(A) Female**	**WT vehicle**	135.5±8.1	271.5±18.8	487.3±23.4	5,046.9±292.5
	***Hfe^−/−^*** ** vehicle**	186.5±12.5‡	273.2±18.1	1,606.3±132.5‡	4,760.0±373.5
	**WT 20 µg LPS**	135.1±14.7	254.8±20.7	508.5±18.2	4,591.1±502.4
	***Hfe^−/−^*** **20 µg LPS**	182.1±3.1⧫	223.1±25.3	1,652.3±125.1⧫	4,808.5±371.6
**(B) Female**	***Hfe^LysMCre^*** ** (− ) vehicle**	139.8±14.6	272.1±17.4	470.2±18.3	5,535.7±728.9
	***Hfe^LysMCre^*** ** (+) vehicle**	156.9±18.0	350.4±25.4	611.3±82.2	6,774.5±624.5
	***Hfe^LysMCre^*** ** (− ) 20 µg LPS**	141.8±8.0	299.1±31.0	514.9±22.2	5,756.3±270.2
	***Hfe^LysMCre^*** ** (+) 20 µg LPS**	161.0±12.6	302.6±10.5	861.9±107.3	6,975.6±605.0

Plasma iron in µg/dL, non-heme tissue iron content in µg iron/g dry tissue.

(A) Female wild-type and *Hfe^−/−^* mice. n = 5–7 per group.

‡ *P*≤0.005 versus WT control mice;

⧫ *P*≤0.001 versus LPS-treated WT mice.

(B) *Hfe^LysMCre^* mice. Vehicle-treated groups: n = 4–5 per group; LPS-treated groups: n = 9–15 per group.

### Alveolar Macrophages of Untreated *Hfe^−/−^* Mice are Iron Depleted and Accumulate Iron in Response to LPS Instillation

**Figure 4:**
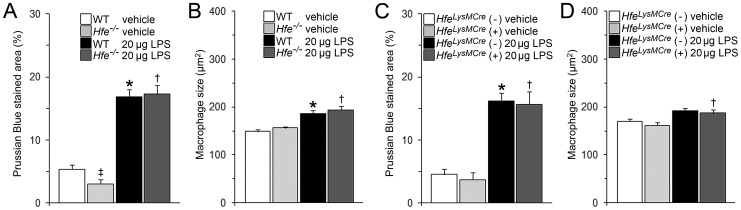
Computerized analysis of cytoplasmic Prussian blue (PB) stained AM. Analysis of iron deposits in AM and cell size as a surrogate parameter for cell activation of AM obtained from female wild-type and *Hfe^−/−^* mice and *Hfe^LysMCre^* mice at 4 h after intratracheal instillation of vehicle or 20 µg LPS. (A) PB-stained iron deposits in AM of female wild-type and *Hfe^−/−^* mice. ^‡^ *P*<0.05 and ^★^ *P*≤0.001 versus WT control mice; ^†^ *P*<0.005 versus *Hfe^−/−^* control mice. (B) AM size in female wild-type and *Hfe^−/−^* mice. n = 5–7 per group. ^★^ *P*≤0.001 versus WT control mice; ^†^ *P*<0.005 versus *Hfe^−/−^* control mice. (C) PB-stained iron deposits in AM of *Hfe^LysMCre^* mice. ^★^ *P*≤0.001 versus *Hfe^LysMCre^* (−) control mice; ^†^ *P*<0.001 versus *Hfe^LysMCre^* (+) control mice. (D) AM size in *Hfe^LysMCre^* mice. n = 4–15 per group. ^†^ *P*<0.05 versus *Hfe^LysMCre^* (+) control mice.

**Figure 5:**
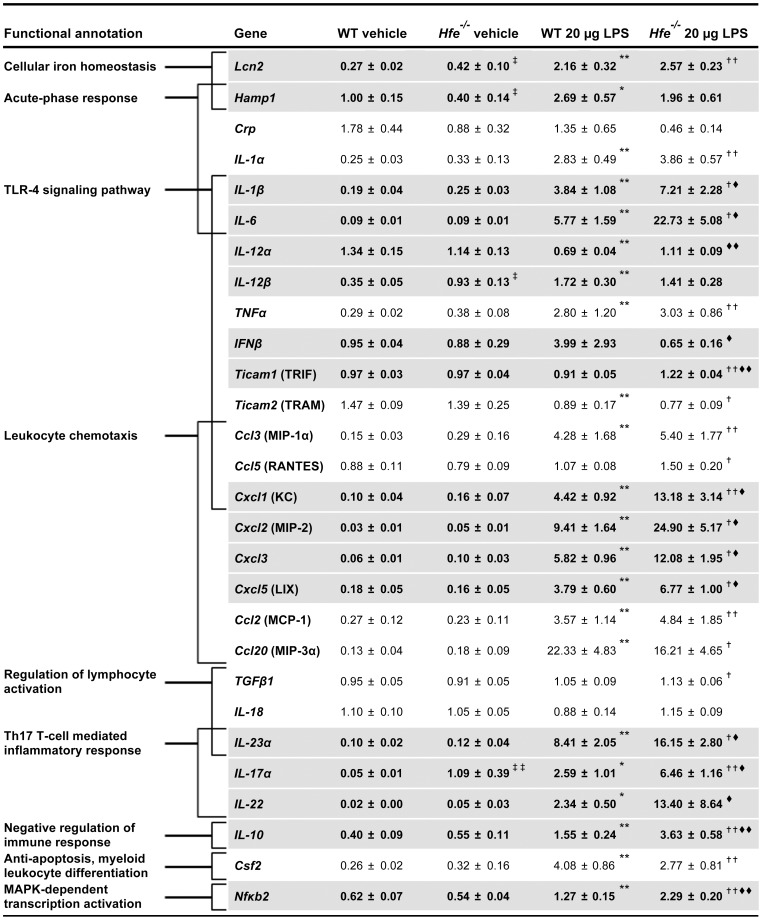
mRNA expression of selected inflammatory mediators in lung samples of female wild-type and *Hfe^−/−^* mice. qPCR results are shown as relative mRNA expression normalized to *GAPDH* mRNA expression. n = 5–7 mice per group. The affiliation to functional annotation groups is indicated by brackets. An overlap of bracket indicates the affiliation of the respective inflammatory mediators to more than one functional annotation group. Genes that differed significantly in expression between wild-type and *Hfe^−/−^* mice in either vehicle- or LPS-treated groups are highlighted in grey and bold letters. ^‡^ *P*<0.05 and ^‡‡^ *P*≤0.001 versus WT control mice; ^★^ *P*<0.05 and ^★★^ *P*≤0.005 versus WT control mice; ^†^ *P*<0.05 and ^††^ *P*≤0.005 versus *Hfe^−/−^* control mice; ^⧫^ *P*<0.05 and ^⧫⧫^ *P*≤0.005 versus LPS-treated WT mice.

Inappropriately low hepatic hepcidin mRNA levels in *Hfe^−/−^* mice (Fig. S2) cause iron depletion of macrophages by deregulating ferroportin levels. To assess whether iron levels are depleted in AM we analyzed intracellular ferric iron deposits by computerized analysis of Prussian blue (PB) staining. We show that AM of female *Hfe^−/−^* mice contained significantly less PB-stained precipitate in the cytoplasm compared to wild-type control mice (Fig. 4A). Our results extend previous observations that show iron depletion in peritoneal macrophages of *Hfe^−/−^* mice [9], [10], [11]. In principle this finding can be explained by low hepatic hepcidin expression (Fig. S2). It is however of interest that pulmonary hepcidin levels are reduced in *Hfe^−/−^* compared to wild-type mice (Fig. 5). Thus, the impairment of a pulmonary hepcidin-controlled ferroportin autoregulatory loop in *Hfe^−/−^* mice may contribute to increased iron export from AM. By contrast, intracellular PB-stained iron deposits in AM of *Hfe^LysMCre^* (+) and *Hfe^LysMCre^* (−) mice did not differ significantly (Fig. 4C), suggesting that macrophage Hfe is not required to control iron levels in AM. Consistent with earlier reports of inflammation-triggered iron sequestration in macrophages [30], [31], LPS-instillation significantly increased the cytoplasmic PB-stained precipitates in AM of wild-type and *Hfe^−/−^* mice, *Hfe^LysMCre^* (−) and *Hfe^LysMCre^* (+) mice (Fig. 4A, 4C). To exclude that differences in the size of the cell area analyzed account for the iron depletion in *Hfe^−/−^* control mice, we additionally measured the cell size of the AM. While the size of the AM of vehicle-treated *Hfe^−/−^* was not different from the one of wild-type mice, we observed a significant increase in AM size in all mouse strains studied following pulmonary LPS instillation. Our finding is consistent with previous observations that relate AM size to inflammatory activity (Fig. 4B, 4D) [32]. Taken together, our analyses suggest that the reduced macrophage iron content in AM of *Hfe^−/−^* mice may contribute to attenuated neutrophil recruitment (Fig. 1). It is further of note that BAL neutrophils of LPS-instilled *Hfe^−/−^* mice showed elevated iron levels compared to LPS-treated wild-type controls (0.54±0.08% vs. 1.10±0.09%; *P*<0.01). To interpret these data measurements of iron levels in BAL neutrophils of untreated mice would be required. However, due to the low numbers of BAL neutrophils in untreated mice accurate assessment of iron levels was not possible.

### *Hfe^−/−^* Mice Show Dysregulated Expression of Inflammatory Mediators in the Lung

To investigate whether *Hfe* deficiency alters gene responses of the lung, we analyzed mRNA expression of selected inflammatory mediators. Genes of interest were preselected based on literature research and by screening data obtained from Iron chip analysis [33] and Affymetrix Mouse Gene 1.0 ST Array (data not shown).

**Figure 6:**
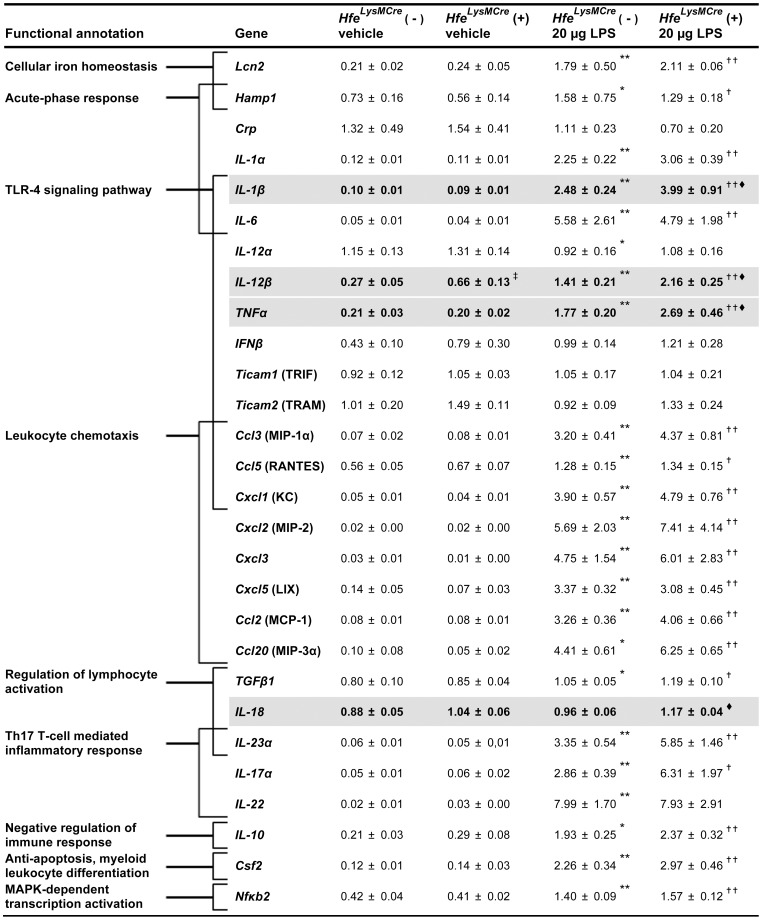
mRNA expression of selected inflammatory mediators in lung samples of female *Hfe^LysMCre^* mice. qPCR results are shown as relative mRNA expression normalized to GAPDH-expression. n = 4–15 mice per group. The affiliation to functional annotation groups is indicated by brackets. An overlap of bracket indicates the affiliation of the respective inflammatory mediators to more than one functional annotation group. Genes that differed significantly in expression between *Hfe^LysMCre^* (−) and *Hfe^LysMCre^* (+) mice in either vehicle- or LPS-treated groups are highlighted in grey and bold letters. ^‡^ *P*<0.05 versus *Hfe^LysMCre^* (−) control mice; ^★^ *P*<0.05 and ^★★^ *P*≤0.005 versus *Hfe^LysMCre^* (−) control mice; ^†^ *P*<0.05 and ^††^ *P*≤0.005 versus *Hfe^LysMCre^* (+) control mice; ^⧫^ *P*<0.05 versus LPS-treated *Hfe^LysMCre^* (−) mice.

Out of 28 genes analyzed 4 genes (hepcidin, *Lcn2*, *IL-12β* and *IL-17α)* showed differential pulmonary mRNA responses in untreated female *Hfe^−/−^* mice compared to wild-type controls. In contrast to reduced hepcidin mRNA expression, lipocalin-2 (*Lcn2*), a putative endogenous iron chelator [11], [17] and a protective factor in allergic airway disease [34], as well as the cytokines, *IL-12β* and *IL-17α* showed increased mRNA expression in untreated *Hfe^−/−^* mice. Altered mRNA expression for hepcidin, Lcn2 and IL-17α seems to be a consequence of *Hfe*-deficiency in cell-types other than macrophages/neutrophils because mRNA expression of these genes is not increased in *Hfe^LysMCre^* (+) compared to *Hfe^LysMCre^* (−) mice (Fig. 6). By contrast, IL-12β mRNA expression is also increased in *Hfe^LysMCre^* (+) mice suggesting alterations in macrophage/neutrophil signaling as a consequence of *Hfe*-deficiency in these cells. However, this alteration was not detectable on protein level (Table 2). In response to pulmonary LPS instillation hepcidin, *Lcn2* and *IL-17α* mRNA levels increased in *Hfe^−/−^* mice compared to vehicle-treated *Hfe^−/−^* mice (Fig. 5).

**Table 2:** Cytokine protein levels in female wild-type, *Hfe^−/−^* and *Hfe^LysMCre^* mice.

	Experimental group		Cxcl1 (KC)		IL-1β		IL-10	IL-12β		
**(A) Female**		**WT vehicle**		146.3±66.9	9.8±0.5	11.6±0.4			64.1±6.6	
		***Hfe^−/−^*** ** vehicle**		239.9±94.0	10.8±0.7	13.3±0.9			75.8±9.4	
		**WT 20 µg LPS**		5,098.3±1,045.0**	19.8±4.0*	13.4±0.9			371.6±62.5**	
		***Hfe^−/−^*** **20 µg LPS**		8,463.2±651.7^††⧫^	19.6±1.9^††^	27.5±7.1† ^⧫^			258.7±48.3^††^	
**(B) Female**		***Hfe^LysMCre^*** ** (− ) vehicle**		123.0±37.8	15.1±3.8	14.1±1.8			55.3±4.2	
		***Hfe^LysMCre^*** ** (+) vehicle**		56.2±7.8	10.9±1.1	13.6±1.3			68.1±18.9	
		***Hfe^LysMCre^*** ** (− ) 20 µg LPS**		5,184.1±569.1*	20.8±2.2	19.9±2.9			181.9±30.1*	
		***Hfe^LysMCre^*** ** (+) 20 µg LPS**		4,832.9±1,129.0†	17.5±2.0†	18.0±4.1			224.1±110.9	

Cytokine protein levels are represented by the fluorescence intensity (FI) as assessed by a Multiplex bead-array based technology assay.

(A) Female wild-type and *Hfe^−/−^* mice. n = 5–7 per group.

★ *P*<0.05 and ^★★^ *P*<0.01 versus WT control mice;

† *P*<0.05 and ^††^ *P*<0.01 versus *Hfe^−/−^* control mice;

⧫ *P*<0.05 versus LPS-treated WT mice. (B) *Hfe^LysMCre^* mice. Vehicle-treated groups: n = 4–5 per group; LPS-treated groups: n = 9–15 per group.

★ *P*<0.01 versus *Hfe^LysMCre^* (−) control mice;

† *P*<0.05 versus *Hfe^LysMCre^* (+) control mice.

Moreover, the response to pulmonary LPS instillation in wild-type and *Hfe^−/−^* mice is hallmarked by differential mRNA expression of chemokines and cytokines involved in TLR4- and Th17-dependent signaling. 25 genes showed increased mRNA expression in either wild-type and/or *Hfe^−/−^* mice (Fig. 5); a response pattern that was qualitatively and quantitatively similar to the one observed in *Hfe^LysMCre^* (+) and *Hfe^LysMCre^* (−) mice (Fig. 6). 12 genes that are functionally linked to pro- or anti-inflammatory responses, showed strikingly higher mRNA expression in LPS-challenged *Hfe^−/−^* compared to wild-type mice (Fig. 5). These included TLR-4 pathway associated cytokines such as *IL-1β* and *IL-6*, CXC-chemokines (e.g. *Cxcl1, Cxcl2, Cxcl3, Cxcl5*), Th17-associated cytokines (e.g. *IL-23α, IL-17α or IL-22*), *Nf-κB2* as well as the anti-inflammatory cytokine *IL-10*. Importantly, mRNA expression of *IFNβ* failed to be induced in LPS-treated *Hfe^−/−^* mice compared to the respective wild-type mice. This was associated with mildly increased mRNA expression of TRIF (*Ticam1*) in *Hfe* ^−/−^ mice (*1.3-fold*), an activator of *IFNβ* mRNA expression [35]. By contrast, mRNA expression of a second *IFNβ* activator located upstream of TRIF, TRAM (*Ticam2*) [35], was downregulated in both *Hfe^−/−^* and wild-type mice following LPS treatment. Interestingly, mRNA expression of a second cytokine expressed in a Mal/MyD88-independent manner, *Ccl5* (RANTES) [35], was not impaired in *Hfe^−/−^* mice (Fig. 5).

To address the question whether differences in cytokine mRNA expression are also observed at the protein level, we measured cytokine expression of *Cxcl1* (KC), *IL-1β, IL-10* and *IL-12β* in the BAL by applying the Multiplex bead-array based technology. We show that protein levels of the pro-inflammatory chemokine *Cxcl1* and anti-inflammatory cytokine IL-10 are significantly increased in the BAL of LPS-treated *Hfe^−/−^* mice compared to the respective wild-type mice. By contrast, a difference in *IL-1β* protein levels was not detectable between *Hfe^−/−^* and wild-type mice 4 h post-treatment (Table 2).

In *Hfe^LysMCre^* (+) mice, only *IL-1β, IL-12β and TNFα* respond with higher mRNA expression compared to *Hfe^LysMCre^* (−) mice upon LPS treatment (Fig. 6). The only gene hyper-induced in both, LPS-treated *Hfe^−/−^* and *Hfe^LysMCre^* (+) mice is *IL-1β* (*1.9 fold and 1.6-fold, respectively*), a cytokine required for neutrophil recruitment in the lung [36]. The corresponding differences in protein levels could however not be detected (Table 2).

To obtain indications for systemic inflammation as a result of pulmonary LPS instillation, we studied the inflammatory gene responses of the liver [26], [27], [28]. The hepatic gene response pattern of wild-type and *Hfe^−/−^* mice following pulmonary LPS instillation was similar to the pulmonary one in that most of the inflammatory genes analyzed responded with increased mRNA expression (Fig. S2 and S3). Similar to the pulmonary response in *Hfe^−/−^* mice, we observed reduced hepcidin and increased *Lcn2* mRNA expression in livers of vehicle-instilled *Hfe^−/−^* mice. Interestingly, hepatic *IFNβ* mRNA expression also failed to be induced upon LPS instillation in *Hfe^−/−^* mice, suggesting a tissue-independent response. Furthermore, several chemokines and cytokines involved in TLR4-dependent signaling were similarly hyper-induced in the liver and the lung of LPS-instilled *Hfe^−/−^* compared to wild-type mice. By contrast, Th17-associated cytokines were not regulated and/or expressed in livers of *Hfe^−/−^* and wild-type mice (Fig. S2).

In *Hfe^LysMCre^* (+) mice hepatic mRNA expression did not overlap with their pulmonary response. Strikingly, the genes differentially expressed in livers of LPS instilled *Hfe^LysMCre^* (+) and *Hfe^LysMCre^* (−) mice (*Ccl2, Ccl3, Cxcl3, Ticam1*) responded with lower mRNA levels in *Hfe^LysMCre^* (+) mice compared to *Hfe^LysMCre^* (−) mice (Fig. S3). This suggests that macrophage *Hfe* is not responsible for the hyper-inducibility of inflammatory genes in *Hfe^−/−^* mice as this alteration is not preserved in livers of *Hfe^LysMCre^* (+) mice. Rather changes in iron homeostasis, such as macrophage iron deficiency that result as a consequence of hepatic *Hfe*-deficiency or the lack of *Hfe* in other cell types seems to modify the inflammatory response. Taken together, we conclude that *Hfe* plays a critical role in regulating the expression of inflammatory mediators.

## Discussion

Competition for iron between pathogens and host can determine the outcome of an infectious disease. Thus, the regulation of cellular and systemic iron metabolism is tightly linked to the immune response. On the one hand, iron levels control the expression of immune-related proteins such as cytokines; on the other hand immune-cell derived mediators and acute phase proteins regulate iron homeostasis [1]. In this study, we provide novel insight into how Hfe, a MHC class I-like protein and critical regulator of systemic iron levels in mice and men controls the local innate immune response of the lung. We show that Hfe is critical for efficient pulmonary neutrophil recruitment following LPS-induced inflammation (Fig. 1). *Hfe* acts specifically on neutrophil influx into the lung as the numbers of circulating blood neutrophils following pulmonary LPS instillation do not differ between *Hfe^−/−^* and wild-type mice (Fig. 2). Attenuated inflammatory responses were previously reported in *Hfe^−/−^* mice following *Salmonella* infection of the peritoneum and gut [9], [10], [11]. Several mechanisms were suggested to cause the attenuated inflammatory response in *Hfe^−/−^* mice including (i) local effects of *Hfe*-deficiency in macrophages or other cell types and (ii) alterations in systemic and/or cellular iron homeostasis that hallmark *Hfe^−/−^* mice.

To determine if local *Hfe*-deficiency in macrophages/neutrophils is involved in the attenuated neutrophil recruitment to the lung in LPS-instilled *Hfe^−/−^* mice we compared effects of LPS instillation in constitutive *Hfe^−/−^* mice and in mice with macrophage/neutrophil-*Hfe^−/−^* deficiency [6]. We demonstrate that selective *Hfe* ablation in macrophages/neutrophils does not cause a significant impairment in LPS-induced neutrophil recruitment, and therefore we propose that *Hfe* in macrophages/neutrophils is unlikely to participate in neutrophil recruitment to the lungs. These results suggest that *Hfe* ablation in other cell types of the lung that are involved in transmigration of neutrophils from the bloodstream, e.g. endothelial cells, fibroblasts and/or alveolar epithelial cells, may play a role in the impaired neutrophil recruitment in *Hfe^−/−^* mice.

We next focused our attention on imbalances of systemic iron homeostasis in *Hfe^−/−^* mice, such as elevated iron levels in plasma and liver (Table 1) and iron depletion in macrophages [4], [6], [37] (Fig. 4A). We show that reduced iron levels also hallmark AM of *Hfe*-deficient mice (Fig. 4A). By contrast, intracellular iron levels are unaltered in AM of *Hfe^LysMCre^* (+) mice, suggesting that low systemic hepcidin levels rather than macrophage Hfe-ablation is responsible for the reduced macrophage iron content. These data contrast previous findings that show reversion of reduced iron levels in macrophages isolated from HH patients by the overexpression of wild-type *Hfe* [38]. In addition, macrophage expressed *Hfe* is dispensable for the control of pulmonary hepcidin mRNA expression [20], which is reduced in lung tissue of *Hfe^−/−^* mice and unaltered in *Hfe^LysMCre^* (+) mice.

The influx of neutrophils into the bronchoalveolar space (first observed 2 h post LPS instillation) is preceded by adhesion of AM to alveolar epithelia, which presents a critical step for neutrophil recruitment [23], [24], [39]. The macrophage iron content may affect this process at multiple steps. For one, macrophage iron depletion can cause an attenuated inflammatory response by impairing TLR-4 signaling [10], a process involved in neutrophil recruitment. Second, the iron content of monocytes controls adhesion to epithelial cells, expression of chemokines and transendothelial migration [40]. We speculate that macrophage iron deficiency in *Hfe^−/−^* mice may diminish their potential for adhesion to lung epithelia, which may contribute to the decelerated recruitment of neutrophils to the alveolar space.

Autocrine and paracrine effects of hepcidin were suggested to contribute to the rapid iron sequestration in peripheral and alveolar macrophages at the site of inflammation [30], [31]. Despite pulmonary hepcidin deficiency and reduced iron levels in macrophages of untreated *Hfe^−/−^* mice, we observed similar levels of intracellular iron accumulation in AM of wild-type, *Hfe^−/−^*, *Hfe^LysMCre^* (−) and *Hfe^LysMCre^* (+) mice in response to LPS treatment. This suggests that the inflammatory response overrides iron-related differences of the steady-state condition. We conclude that the reduced iron content in macrophages of *Hfe^−/−^* mice will only play a role in diminished neutrophil recruitment, if the initial LPS contact primes the inflammatory response of the macrophage in an iron-dependant manner that is not reversible by the subsequent iron accumulation.

In addition to low macrophage iron content, *Hfe^−/−^* mice are characterized by increased plasma and liver iron levels. Earlier studies indicate that iron overload inhibits transendothelial migration and cell adhesion of neutrophils [41] and affects the expression of adhesion molecules, such as L-selectin, E-selectin and ICAM-1 [42]. Importantly, neutrophils obtained from patients with HH or other iron overload diseases such as Thalassemia major showed impaired chemotaxis, phagocytosis and/or bactericidal activity [43], [44]. Whether or not this is caused by alterations of iron homeostasis in neutrophils has not been analyzed. However, a single report demonstrates that neutrophils are capable of secreting hepcidin under inflammatory conditions [45]. Here we show that BAL neutrophils contain significantly lower amounts of iron compared to macrophages but that iron accumulates in LPS-instilled *Hfe^−/−^* mice compared to LPS-instilled wild-type mice. Putative implications of this finding need to be addressed by future experiments.

Taken together, we conclude that imbalances in iron homeostasis (both macrophage iron deficiency as well as elevated plasma iron levels) likely affect cell adhesion and transendothelial migration in a complex manner. Based on studies discussed above we believe that deregulation of cell adhesion and transendothelial migration contributes to the observed attenuation of neutrophil recruitment in *Hfe^−/−^* mice. Further studies will be required to address this question.

Previous work [9], [10] relates the attenuated recruitment of innate immune cells in *Hfe^−/−^* mice in response to *Salmonella* or LPS treatment to impairment in TRAM/TRIF-signaling. As a consequence *IFNβ* mRNA expression and/or *TNFα* and *IL-6* protein secretion is reduced in cultured macrophages. Decreased *TNFα* secretion was also described in monocytes from HH patients following LPS stimulation [15]. By contrast, no differences in cytokine production were identified in a second study of *Salmonella*-induced infection [11] and following intraperitoneal LPS-administration [16] in *Hfe^−/−^* mice. A major finding of this study is that *Hfe^−/−^* mice display attenuated neutrophil recruitment upon LPS stimulation compared to wild-type mice despite of increased mRNA expression and protein secretion of Mal/MyD88-dependent cytokines/chemokines (Fig. 5, Table 2) [35]. Several possibilities may explain this finding: (i) given that certain cytokines such as *IL-6* and *IFNβ* possess both pro- and anti-inflammatory functions [46], [47], [48] it is conceivable that in balance an anti-inflammatory cytokine effect predominates in *Hfe^−/−^* mice; (ii) *IFNβ*, which fails to be stimulated in *Hfe^−/−^* mice upon pulmonary LPS instillation may play a so far unidentified role for the recruitment of neutrophils to the site of inflammation; (iii) cytokines/chemokines may be hyper-induced in the alveolar epithelia of *Hfe^−/−^* mice in response to the inadequately low neutrophil recruitment compensating for the presumably impaired TLR4 signaling in AM [9], [10]. Such a feedback loop is conceivable, as alveolar epithelial type II (AEII) or endothelial cells are known to actively participate in the inflammatory response and the recruitment of neutrophils [49], [50], [51], [52]. Excessive TLR4 signaling in these cell types however fails to recruit adequate numbers of neutrophils to the BAL. Our data thus support previous results showing that TLR4 expression solely on bone-marrow derived cells is required to initiate pulmonary neutrophil recruitment following LPS instillation [53].

Increased intracellular iron levels in macrophages are associated with impaired *IFN*γ-mediated expression of proinflammatory cytokines such as *TNFα* and *IL-1β* [54], [55], [56]. By contrast, an exacerbated hepatic and splenic proinflammatory condition in response to LPS treatment was recently described in iron deficient mice [57] and iron-deficient AM [58]. These findings are suggestive that reduced iron levels in AM of *Hfe^−/−^* mice may contribute to the increased mRNA and protein levels of some cytokines (Fig. 5, Table 2). As AM constitute a numerically inferior cell population in the lung it is however likely that other cell types will contribute to the overall gene response patterns observed. Whatever the reason for the excessive production of cytokines/chemokines, they fail to compensate for the defect imposed by the lack of *Hfe*.

Finally, enhanced mRNA expression of *Lcn2* in *Hfe^−/−^* mice was proposed to cause an attenuated inflammatory response in the liver and the spleen of *Hfe^−/−^* mice [11]. In this study we also observe significantly increased *Lcn2* mRNA expression in lungs (and livers) of untreated *Hfe^−/−^* mice compared to wild-type controls. Whether increased *Lcn2* levels can modulate pulmonary neutrophil recruitment needs to be established in future work.

In conclusion, our results reveal a hitherto unknown interlink between iron homeostasis and pulmonary inflammation, and provide novel insight into *in vivo* consequences of *Hfe*-deficiency in the lung. The underlying molecular mechanisms are likely multifactorial and include elevated systemic iron levels, alveolar macrophage iron deficiency and hitherto unexplored functions of Hfe in resident pulmonary cell types. As a consequence, pulmonary cytokine expression is out of balance and neutrophils fail to be recruited efficiently to the bronchoalveolar compartment, a process required to protect the host from infections. Little is known about the frequency of pulmonary infections in HH patients. Because neutrophils are an important first line of defense against bacterial pathogens, our results in a murine disease model suggest that HH may be associated with increased susceptibility to bacterial infections of the respiratory system. Further, we speculate that Hfe may be implicated in bacterial host defense, as well as acute and chronic neutrophilic inflammatory processes of the lung in subjects without HH, and may thus serve as a novel target for anti-infective and anti-inflammatory therapies for common lung diseases including pneumonia, acute respiratory distress syndrome (ARDS), chronic obstructive pulmonary disease (COPD) and cystic fibrosis.

## Materials and Methods

### Ethics Statement

All mouse breeding and animal experiments were approved by the Animal Care and Use Committee of the Regierungspräsidium Karlsruhe, Germany and conducted in compliance with the guidelines of the Institutional Animal Care and Use Committee of the European Molecular Biology Laboratory.

### Mice

*WT, Hfe^−/−^* and *Hfe^LysMCre^* mice used in this study have previously been described [6], [37], [59]. Age- and sex-matched cohorts were studied at 14–19 weeks of age.

### Experimental Protocol

Acute pulmonary inflammation was induced by a single intratracheal instillation of LPS. Mice were anaesthesised by inhalation of isoflurane and received either 1 µg or 20 µg LPS (*Escherichia coli* 055:B5, Sigma, St. Louis, MO) in 20 µl sterile PBS (PBS 1×pH 7.4, Gibco-Invitrogen). Control animals received the same volume of sterile PBS.

4 h after exposure, mice were anaesthetized with an intraperitoneal injection of ketamine (120 mg/kg) and xylazine (16 mg/kg). Heparinized blood for hematological and plasma analysis was collected from the inferior vena cava and mice were sacrificed by exsanguination. Hematologic analysis and white blood cell counts were analyzed at the University Medical Center, Heidelberg. The bronchoalveolar lavage (BAL) was performed on the left lung lobe with 0.5×0.035 ml/g body weight of cold sterile PBS in two consecutive washes and a recovery of ≥60% was considered acceptable [60]. Right lung lobes, liver, spleen and duodenum were collected, immediately fresh-frozen in liquid nitrogen and stored at −80°C for subsequent analyses.

### Preparation of Bronchoalveolar Lavage for Analysis

The recovered BAL fluid was centrifuged and the cell free supernatant was stored at −80°C. Total cell counts were determined using a haemocytometer and differential cell counts were determined in cytospin preparations stained with May Grünwald-Giemsa as described [60] or 9% potassium ferrocyanide solution [Prussian Blue stain; PB] for analysis of intracellular iron deposits.

### Determination of Macrophage Size and Intracellular Iron Content

Digital true color (RGB) images of PB-stained cytospin slides and negative controls were acquired at 400×magnification with a resolution of 300×300 dpi using an Olympus IX71 inverted microscope (Olympus, Center Valley, PA) and Cel
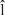
F software (v2.7, Olympus Soft Imaging Solutions GmbH, Münster, Germany) at constant illumination. Images were not corrected for brightness, contrast or other parameters prior to analysis.

The total area of BAL alveolar macrophages was determined as a surrogate parameter for cell activation [32]. The mean area was determined from a minimum of 80 macrophages per animal by the Cel
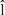
F software and expressed in µm^2^ as previously described [32]. Intracellular iron deposits of intact alveolar macrophages were determined using ImagePro Plus (Media Cybernetics, Version 5.1.0)**.** The color spectrum threshold of the intracellular hemosiderin deposits was set manually and maintained at a fixed value for all experimental groups as well as negative controls. Intracellular ferritin and hemosiderin deposits in the cytoplasm were calculated as the area of interest in the circumscribed total area of macrophage minus the cell nucleus. Mean iron content was determined from a minimum of 50 cells per animal and expressed in % of mean cytoplasmatic area. Intracellular iron content measurements were performed by an investigator blinded to the genotype and treatment of the mice (manuscript in preparation). An analogous experimental approach was applied to determine intracellular iron levels of BAL neutrophils.

### Determination of Tissue Non-heme Iron Content and Plasma Iron Concentration

Non-heme iron content of liver, spleen and lung tissues was determined by the modified method of Torrance and Bothwell [61] as described previously [37]. Values are expressed as µg of iron per gram of dry tissue. Iron concentration in plasma samples was measured as previously described [6].

### RNA Extraction, QRT-PCR

Total RNA was isolated from snap-frozen liver and right-lobe lung tissues using the TRIzol reagent (Invitrogen, Carlsbad, CA). 1–2 µg of total RNA were reverse transcribed using SuperScript II Reverse Transcriptase (Invitrogen) according to the manufacturer’s instructions.

Quantitative Real-time PCR was carried out in a 20 µl reaction volume using the SYBR Green I dye on ABI Prism 7500 (Applied Biosystems, Foster City, CA). Primer sequences are given in Table S1. Expression level of target genes was normalized by the expression level of the endogenous reference gene *GAPDH* by using the modified Pfaffl formula [62].

### Measurement of Cytokine Protein Expression in BAL Supernatants

Protein levels of *Cxcl1* (KC), *IL-1β*, *IL-10* and *IL-12β* were determined in BAL supernatants applying Multiplex bead-array based technology. Measurements were performed on a BioPlex 200 System using the Bio-Plex Pro Cytokine Reagent Kit and Bio-Plex Pro Mouse Cytokine sets for respective cytokines (Bio-Rad, Hercules, CA) according to manufacturer’s instructions. Cytokine protein levels are given as fluorescence intensity.

### Statistical analysis

All data were analyzed using the SigmaStat version 3.1 (Systat Software, Erkrath, Germany) and given as mean ± SEM. Statistical analyses were performed using Student’s t-test and Mann-Whitney Rank Sum test as appropriate, and *P*<0.05 was accepted as significant.

## Supporting Information

Figure S1 **Circulating neutrophil levels (in cells/nL) in** ***Hfe^LysMCre^*** **mice.** Instillation of vehicle (n  = 4–5 per group) or 20 µg LPS (n = 9–15 per group). ^★^ *P*<0.05 versus *Hfe^LysMCre^* (−) control mice; ^†^ *P*≤0.005 versus *Hfe^LysMCre^* (+) control mice.
(TIF)

Figure S2 **mRNA expression of selected inflammatory mediators in liver samples of female wild-type and** ***Hfe^−/−^*** **mice.** qPCR-results are given as relative expression normalized to GAPDH-expression. n = 5–7 mice per group. Affiliation to functional annotation groups is demonstrated by brackets. Overlapping of brackets symbolizes affiliation of respective inflammatory mediators to more than one functional annotation group. Genes that differed significantly in expression between wild-type and *Hfe^−/−^* mice in either vehicle- or LPS-treated groups are highlighted in grey and bold letters. ^‡^ *P*<0.05 versus WT control mice; ^★^ *P*<0.05 and ^★★^ *P*≤0.005 versus WT control mice; ^†^ *P*<0.05 and ^††^ *P*≤0.005 versus *Hfe^−/−^* control mice; ^⧫^ *P*<0.05 and ^⧫⧫^ *P*≤0.005 versus LPS-treated WT mice.
(TIF)

Figure S3 **mRNA expression of selected inflammatory mediators in liver samples of female** ***Hfe^LysMCre^*** **mice.** qPCR-results are given as relative expression normalized to GAPDH-expression. n = 4–15 mice per group. Affiliation to functional annotation groups is demonstrated by brackets. Overlapping of brackets symbolizes affiliation of respective inflammatory mediators to more than one functional annotation group. Genes that differed significantly in expression between *Hfe^LysMCre^* (−) and *Hfe^LysMCre^* (+) mice in either vehicle- or LPS-treated groups are highlighted in grey and bold letters. ^‡^ *P*<0.05 versus *Hfe^LysMCre^* (−) control mice; ^★^ *P*<0.05 and ^★★^ *P*≤0.005 versus *Hfe^LysMCre^* (−) control mice; ^†^ *P*<0.05 and ^††^ *P*≤0.005 versus *Hfe^LysMCre^* (+) control mice; ^⧫^ *P*<0.05 versus LPS-treated *Hfe^LysMCre^* (−) mice.
(TIF)

Table S1 Primer sequences of selected genes analyzed by qPCR.
(DOC)
