# Supplementary figures and images for: Hfe Deficiency Impairs Pulmonary Neutrophil Recruitment in Response to Inflammation
Source: PLoS One. 2012 Jun 21;7(6):e39363. doi: 10.1371/journal.pone.0039363 (PMC3383765; doi:10.1371/journal.pone.0039363)

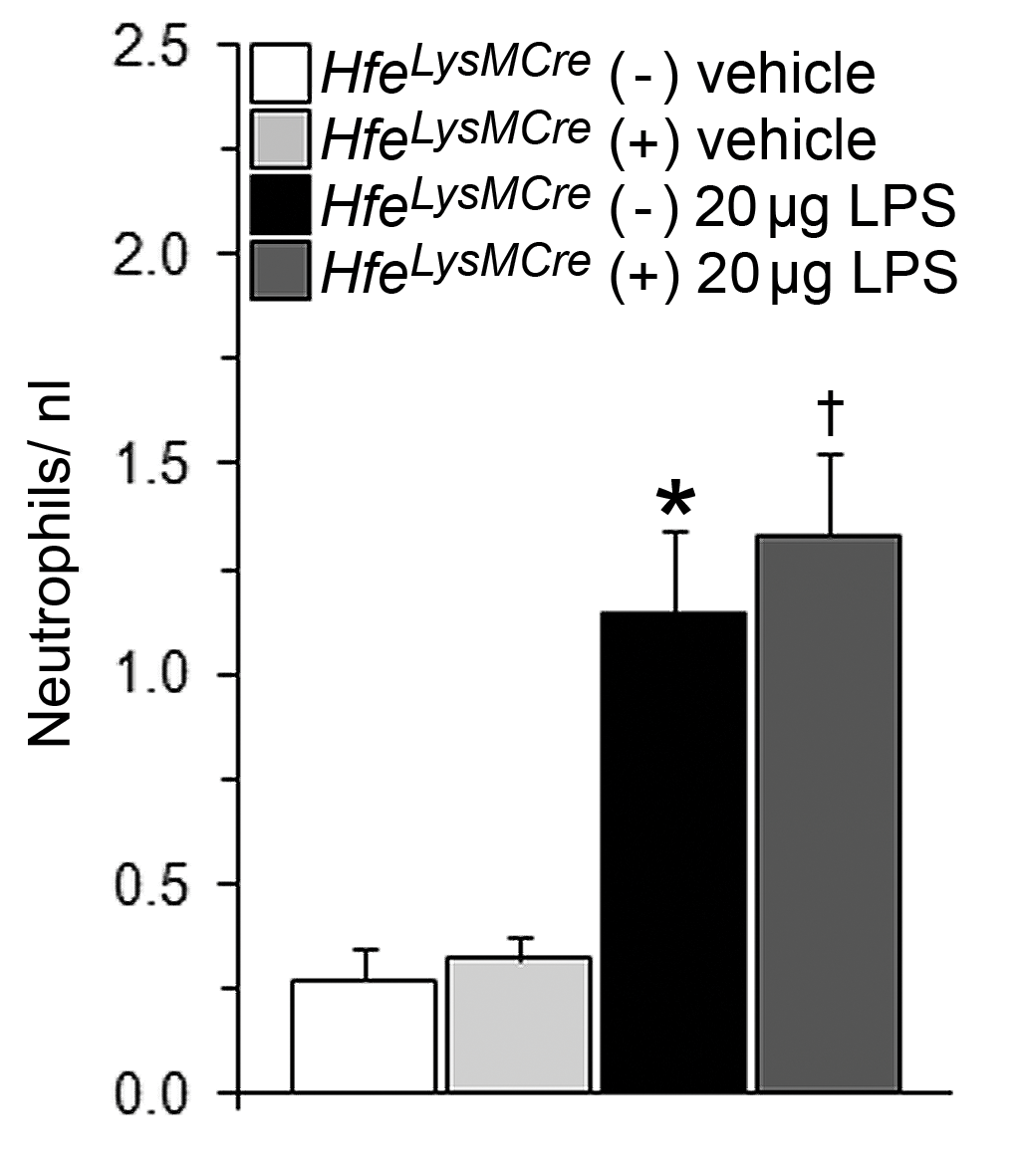

Supplement: Figure S1 — Circulating neutrophil levels (in cells/nL) in HfeLysMCre mice. Instillation of vehicle (n = 4–5 per group) or 20 µg LPS (n = 9–15 per group). ★ P<0.05 versus HfeLysMCre (−) control mice; † P≤0.005 versus HfeLysMCre (+) control mice. (TIF) [file pone.0039363.s001.tif]

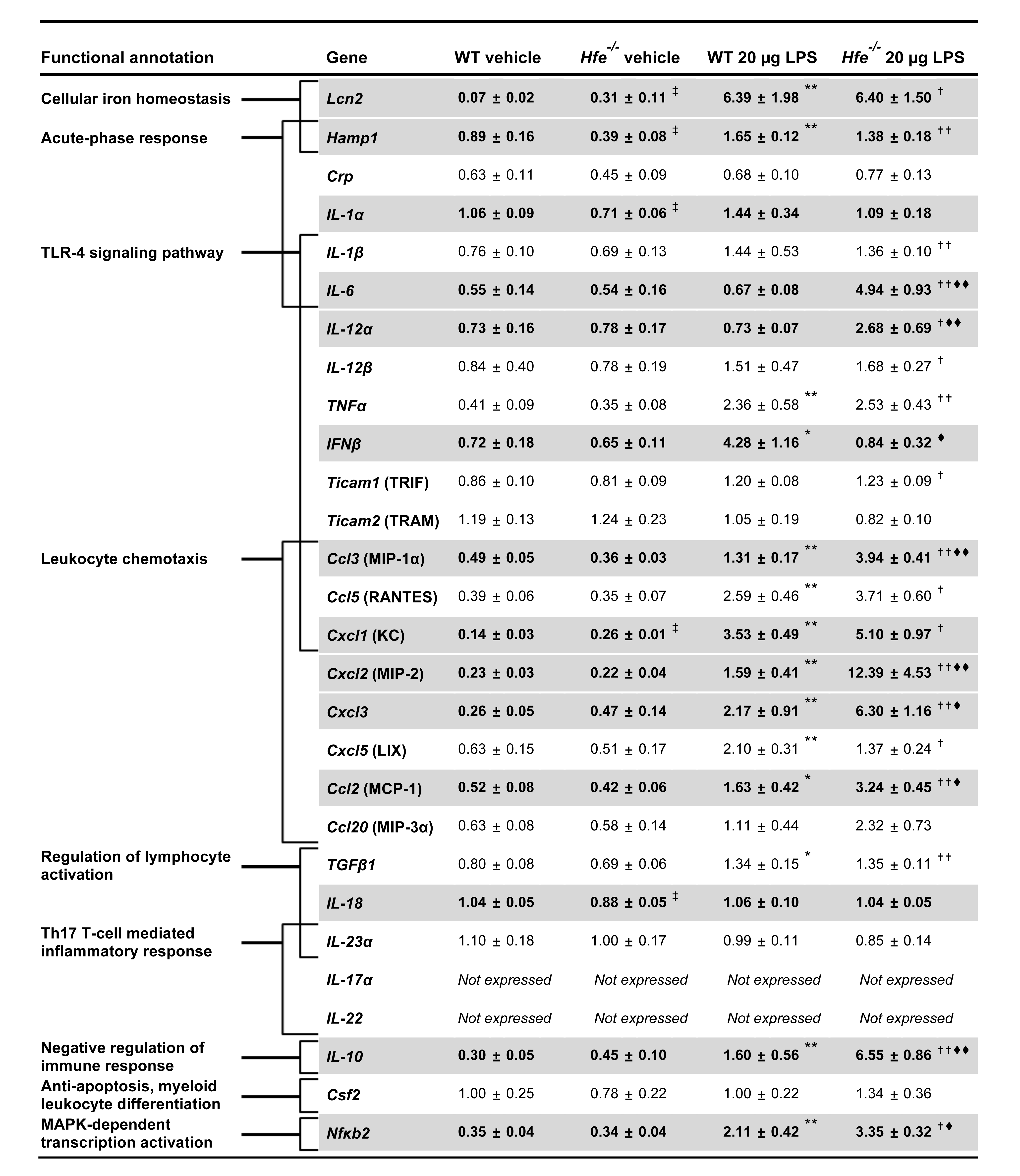

Supplement: Figure S2 — mRNA expression of selected inflammatory mediators in liver samples of female wild-type and Hfe−/− mice. qPCR-results are given as relative expression normalized to GAPDH-expression. n = 5–7 mice per group. Affiliation to functional annotation groups is demonstrated by brackets. Overlapping of brackets symbolizes affiliation of respective inflammatory mediators to more than one functional annotation group. Genes that differed significantly in expression between wild-type and Hfe−/− mice in either vehicle- or LPS-treated groups are highlighted in grey and bold letters. ‡ P<0.05 versus WT control mice; ★ P<0.05 and ★★ P≤0.005 versus WT control mice; † P<0.05 and †† P≤0.005 versus Hfe−/− control mice; ⧫ P<0.05 and ⧫⧫ P≤0.005 versus LPS-treated WT mice. (TIF) [file pone.0039363.s002.tif]

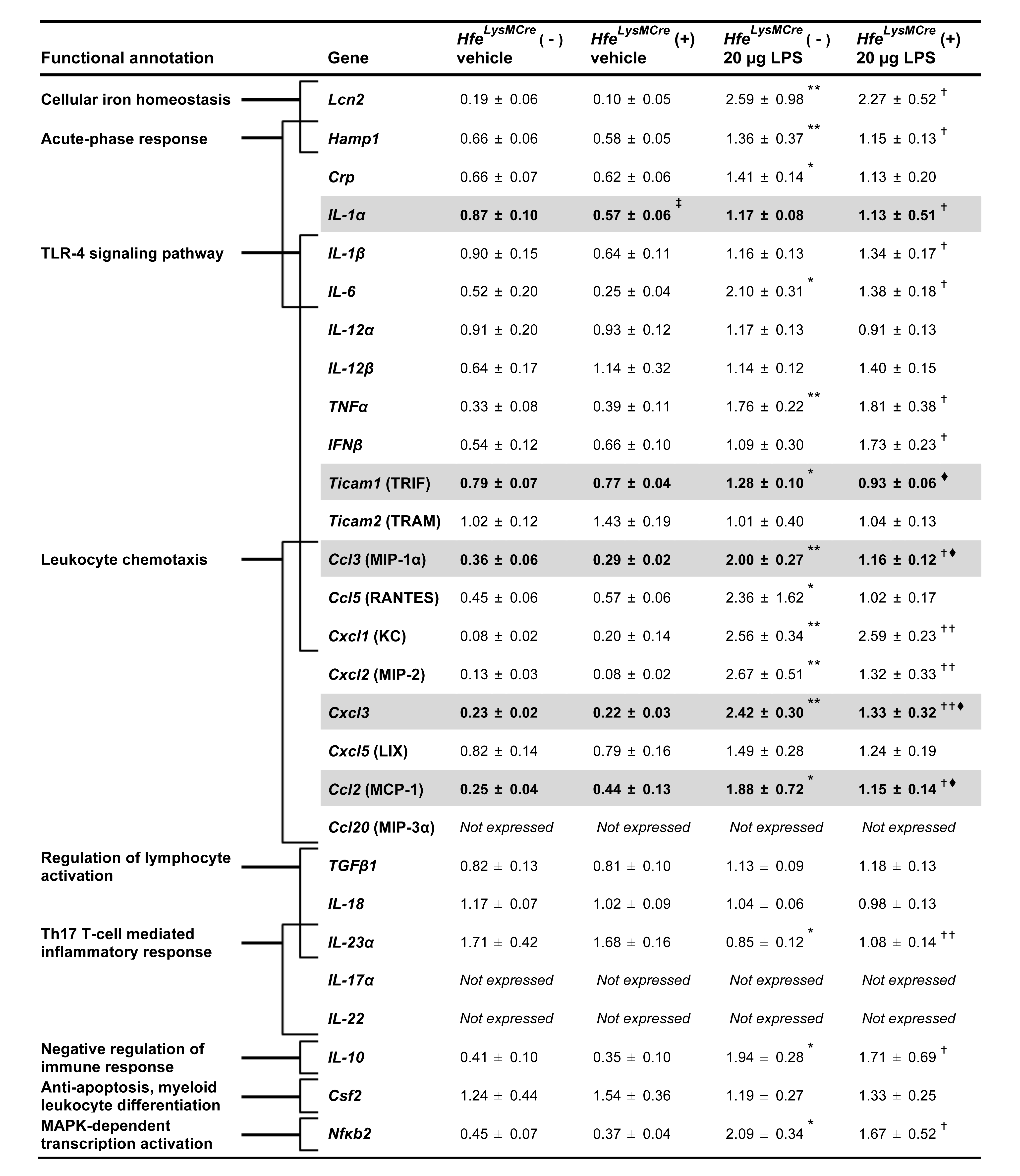

Supplement: Figure S3 — mRNA expression of selected inflammatory mediators in liver samples of female HfeLysMCre mice. qPCR-results are given as relative expression normalized to GAPDH-expression. n = 4–15 mice per group. Affiliation to functional annotation groups is demonstrated by brackets. Overlapping of brackets symbolizes affiliation of respective inflammatory mediators to more than one functional annotation group. Genes that differed significantly in expression between HfeLysMCre (−) and HfeLysMCre (+) mice in either vehicle- or LPS-treated groups are highlighted in grey and bold letters. ‡ P<0.05 versus HfeLysMCre (−) control mice; ★ P<0.05 and ★★ P≤0.005 versus HfeLysMCre (−) control mice; † P<0.05 and †† P≤0.005 versus HfeLysMCre (+) control mice; ⧫ P<0.05 versus LPS-treated HfeLysMCre (−) mice. (TIF) [file pone.0039363.s003.tif]
