# Supplementary material for: Hfe Deficiency Impairs Pulmonary Neutrophil Recruitment in Response to Inflammation
Source: PLoS One. 2012 Jun 21;7(6):e39363. doi: 10.1371/journal.pone.0039363 (PMC3383765; doi:10.1371/journal.pone.0039363)
Supplement: Table S1 — Primer sequences of selected genes analyzed by qPCR. (DOC) [file pone.0039363.s004.doc]

**Table S1** Primer sequences of selected genes analyzed by qPCR.

| **Gene** | **Accession** |  | **Primer sequence** |
| --- | --- | --- | --- |
| **Gapdh** | NM_008084.2 | Fwd | 5' - TGTCCGTCGTGGATCTGAC - 3' |
|  |  | Rev | 5' - CCTGCTTCACCACCTTCTTG - 3' |
| **Ccl2** | NM_011333.3 | Fwd | 5' - CATCCACGTGTTGGCTCA - 3' |
|  |  | Rev | 5' - GATCATCTTGCTGGTGAATGAGT - 3' |
| **Ccl3** | NM_011337.2 | Fwd | 5' - TGCCCTTGCTGTTCTTCTCT - 3' |
|  |  | Rev | 5' - GTGGAATCTTCCGGCTGTAG - 3' |
| **Ccl5** | NM_013653.3 | Fwd | 5' - TGCAGAGGACTCTGAGACAGC - 3' |
|  |  | Rev | 5' - GAGTGGTGTCCGAGCCATA - 3' |
| **Ccl20** | NM_016960.2 | Fwd | 5' - AACTGGGTGAAAAGGGCTGT - 3' |
|  |  | Rev | 5' - GTCCAATTCCATCCCAAAAA |
| **Crp** | NM_007768.4 | Fwd | 5' - TCAGCTTCTCTCGGACTTTTG - 3' |
|  |  | Rev | 5' - AGGTGTTCAGTGGCTTCTTTG - 3' |
| **Csf2** | NM_009969.4 | Fwd | 5' - GCATGTAGAGGCCATCAAAGA - 3' |
|  |  | Rev | 5' - CGGGTCTGCACACATGTTA - 3' |
| **Cxcl1** | NM_008176.3 | Fwd | 5' - ACTCCAACACAGCACCATGA - 3' |
|  |  | Rev | 5' - TGGTCTGCAGGCACTGAC - 3' |
| **Cxcl2** | NM_009140.2 | Fwd | 5' - CCTGGTTCAGAAAATCATCCA - 3' |
|  |  | Rev | 5' - CTTCCGTTGAGGGACAGC - 3' |
| **Cxcl3** | NM_203320.2 | Fwd | 5' - GCCACACTCCAGCCTAGC - 3' |
|  |  | Rev | 5' - GCCACAACAGCCCCTGTA - 3' |
| **Cxcl5** | NM_009141.2 | Fwd | 5' - GAAAGCTAAGCGGAATGCAC - 3' |
|  |  | Rev | 5' - TTCACTGGGGTCAGAGTCCT - 3' |
| **Hamp1** | NM_032541.1 | Fwd | 5' - ATACCAATGCAGAAGAGAAGG - 3' |
|  |  | Rev | 5' - AACAGATACCACACTGGGAA - 3' |
| **Ifnb1** | NM_010510.1 | Fwd | 5' - CAGCTCCAAGAAAGGACGAAC - 3' |
|  |  | Rev | 5' - GGCAGTGTAACTCTTCTGCAT - 3' |
| **Il1a** | NM_010554.4 | Fwd | 5' - TTGGTTAAATGACCTGCAACA - 3' |
|  |  | Rev | 5' - GAGCGCTCACGAACAGTTG - 3' |
| **Il1b** | NM_008361.3 | Fwd | 5' - GCAACTGTTCCTGAACTCAACT - 3' |
|  |  | Rev | 5' - ATCTTTTGGGGTCCGTCAACT - 3' |
| **Il6** | NM_031168.1 | Fwd | 5' - GCTACCAAACTGGATATAATCAGGA - 3' |
|  |  | Rev | 5' - CCAGGTAGCTATGGTACTCCAGAA - 3' |
| **Il10** | NM_010548.2 | Fwd | 5' - CAGAGCCACATGCTCCTAGA - 3' |
|  |  | Rev | 5' - TGTCCAGCTGGTCCTTTGTT - 3' |
| **Il12a** | NM_008351.2 | Fwd | 5' - CCATCAGCAGATCATTCTAGACAA - 3' |
|  |  | Rev | 5' - CGCCATTATGATTCAGAGACTG - 3' |
| **Il12b** | NM_008352.2 | Fwd | 5' - ATCGTTTTGCTGGTGTCTCC - 3' |
|  |  | Rev | 5' - GGAGTCCAGTCCACCTCTACA - 3' |
| **Il17a** | NM_010552.3 | Fwd | 5' - TGTGAAGGTCAACCTCAAAGTCT - 3' |
|  |  | Rev | 5' - GAGGGATATCTATCAGGGTCTTCAT - 3' |
| **Il18** | NM_008360.1 | Fwd | 5' - CAACCTTCCAAATCACTTCCT - 3' |
|  |  | Rev | 5' - TCCTTGAAGTTGACGCAAGA - 3' |
| **Il22** | NM_016971.2 | Fwd | 5' - TTTCCTGACCAAACTCAGCA - 3' |
|  |  | Rev | 5' - CGCCTTGATCTCTCCACTCT - 3' |
| **Il23a** | NM_031252.2 | Fwd | 5' - CACCAGCGGGACATATGAA - 3' |
|  |  | Rev | 5' - CCTTGTGGGTCACAACCAT - 3' |
| **Lcn2** | NM_008491.1 | Fwd | 5' - CCATCTATGAGCTACAAGAGAACAAT - 3' |
|  |  | Rev | 5' - TCTGATCCAGTAGCGACAGC - 3' |
| **Nfkb2** | NM_019408.3 | Fwd | 5' - TGGAACAGCCCAAACAGC - 3' |
|  |  | Rev | 5' - CACCTGGCAAACCTCCAT - 3' |
| **Tgfb1** | NM_011577.1 | Fwd | 5' - TGGAGCAACATGTGGAACTC - 3' |
|  |  | Rev | 5' - CAGCAGCCGGTTACCAAG - 3' |
| **Tnf** | NM_013693.2 | Fwd | 5' - TGCCTATGTCTCAGCCTCTTC - 3' |
|  |  | Rev | 5' - GAGGCCATTTGGGAACTTCT - 3' |
| **Ticam1** | NM_174989.4 | Fwd | 5' - AACCTCCACATCCCCTGTTTT - 3' |
|  |  | Rev | 5' - GCCCTGGCATGGATAACCA - 3' |
| **Ticam2** | NM_173394.3 | Fwd | 5' - TGGTCAAGCAGTACCACTTCC - 3' |
|  |  | Rev | 5' - GAGACGCCTTAGCCTCCAGT - 3' |
